# New York State Medical Society, Fifty-Seventh Session

**Published:** 1864-03

**Authors:** 


### NEW YORK STATE MEDICAL SOCIETY, FIFTY-SEVENTH SESSION.

The 57th Session of the New York State Medical Society, organized Tuesday, Feb 2d, at eleven o'clock, in the Supervisors' room of the City Hall. Dr. Daniel P. Bissell, of Utica, President, in the chair. The session was opened with prayer by the Rev. Dr. Wykoff. After which the President delivered in a brief and very appropriate manner, his Inaugural Address. Among the important points suggested in this address were, that hereafter two Vice-Presidents be chosen, and that, in view of the onerous duties attached to the position of Secretary of this Society, a salary be connected with his office.

The Committee on the Merit Cash Prize Essay, reported that they have received three papers on the subject presented, namely, "How complete is the Protection of Vaccination, and what are the dangers of Communicating other Diseases with Vaccinia?" They have read them all carefully, and while each contains much that is valuable, the one marked 101 is considered a very superior paper, and its circulation calculated to do good. The Committee are unanimous in recommending that paper for the prize. Agreeably to the suggestion of the Secretary, and after consultation with other members of the Society, the Committee have consented to present in their report a competitive subject for the coming year, hoping that its early announcement will secure a wider competion. The subject chosen is the following: "The Pathology and Treatment of Chronic Diarrhœa, contracted in camps and Malarious Regions," illustrated by cases; competitors must be citizens of this State, and they are required to hand in to either of the Committee their essays by the 15th of December, 1864, at latest, and as much earlier as convenient, that sufficient time may be given for examination and consultation.

The report was accepted, and the Secretary broke the seal, and announced Dr. A. N. Bell, of Brooklyn, as the successful essayist,

It was then resolved to append to the essay of Dr. Bell the paper on vaccination, published by the U. S. Sanitary Commission.

Dr. Percy gave an abstract of a paper prepared by him for the Society, on "The Food of Cities." The matter principally brought before the Society was the diseased condition and innutritious qualities of the milk and meats, from animals fed at the distillery dairies. He gave many instances of disease produced in individuals by this mik, and cases of immediate sickness from swill-fed beef. He also mentioned cases of malignant pustule, and evident invasion of *Trichini spiralis* in swill-fed beef. The remarks made by him brought out several other members, giving their experience on this subject.

On motion, it was resolved,

That a Committee of three be appointed by this Society, to request the State Legislature to appoint a Committee, to meet with them, and devise some law that will completely put a stop to the sale of swill milk.

Dr. Furman presented the following from the New York County Medical Society:

"At a regular meeting of the New York County Medical Society, held January 4, 1864, the following resolution was passed:—

"*Resolved.*—That in view of the unsettled state of opinion among medical practitioners, concerning the propriety of advertising "Specialities" in medical or other journals. the delegates of this society be instructed to bring this subject before the Medical Society of the State of New York at its next meeting, with a view to the establishment of some definite regulations concerning it."

As one of the delegates of that Society, Dr. F. moved that a Committee be appointed to consider this subject and report before the adjournment of this meeting; the chair appointed as such Committee, Drs. Brinsmade, Townsend and Furman.

Dr. Brinsmade presented the following report and resolutions:

The undersigned, appointed a Special Committee to report upon a resolution passed by the Medical Society of the county of New York, in relation to the propriety of medical practitioners advertising their "Specialty" in medical or other journals, and referred to this Society for decision, beg leave to offer the following resolutions:

*Resolved,* That in the opinion of this Society it is impossible to define the limits of advertising "Medical Specialties," either in medical or other journals.

*Resolved,* That advertisements indicating location and residence, are the utmost limits of self-announcement, consistent with professional dignity; and that all reference to special branches of medical practice, as extra inducements to patronage, should be deemed violations of the code of medical ethics.

*Resolved,* That hereafter any medical practitioner, so offending, shall be deemed disqualified as a Delegate to or for membership of this Society; and if already a delegate to or member thereof, shall be deemed a fit subject for discipline.

*Resolved,* That this Society recommends all medical societies in the State of New York to adopt the foregoing resolutions, with a view to establish the true dignity of our profession.

*Resolved,* That the foregoing resolutions be transmitted to the American Medical Association at its next annual meeting, as an expression of the opinion of the Medical Society of the State of New York, and, that for the purpose a Committee of Presentation be appointed.

Signed: THOS. C. BRINSMADE,
HOWARD TOWNSEND,
GUIDO FURMAN.

The report was accepted, and on motion of Dr. Jenkins, the subject was made the special order for the second day of the next annual meeting at twelve M.

Dr. Orton, Secretary of the Nominating Committee, presented that Committee's report, and the Society elected the following officers:

*President*—Fred. Hyde, M. D.

*Vice-President*—George J. Fisher, M. D.

*Secretary*—S. D. Willard, M. D.

*Treasurer*—J. V. P. Quackenbush, M. D.

*Committee of Publication*—Drs. S. D. Willard, S. O. Vanderpoel, Samuel H. Freeman.

*Censors*—Southern District: Drs. H. D. Bulkley, N. C, Husted, John Ball. Eastern Disrrict: Drs. B. P. Staats, T. C. Brinsmade, Peter McNaughton, Middle District: Drs. M. M. Bragg, C. B, Coventry, A. F. Doolittle. Western District: Drs. Alex. Thompson, C. M. Crandall,, Edward Hall.

The following is a list of the gentlemen's names registered:

Drs. D. P. Bissell, Joel Foster, S. D. Willard, C. E. Van Anden, N. C. Husted Guido Furman, Wm. Govan, M. C. Hasbrouck, J. O. Cobb, F. Tourtletot, A Haun, A. L. Saunders, John E. Todd, J. F. Jenkins, J. Towler, E. S. Lyman, Jas. Kennedy, Jas. Ferguson, Jas. Lee, Fred. Hyde, B. P. Staats, E. H. Parker, H. K. Willard, H. H. S. Crandall, A. F. Doolittle, Geo. W. Bradford, Alden March, P. V. N. Morris. Louis Elsberg, Jas. Whitford, A. J. Dallas, D. B. Whitney, J. King Merritt, W. L. Appley, C. M. Crandall, J. H. Armsby, John Ferguson, Hiram Corliss, J. G. Orton, T. C. Brinsmade, S. O, Vanderpoel, T. W. Blatchford, A. D. Hull, Howard Townsend, John Swisburne, J. T. Williams, R. L. Allen, E. R. Squibb. J. S. Weidman, D. W. Burdich, R. Blawis, S H. Harrington, J. K. Chamberlayne, J. Newkirk, E. L. Beadle, H. S. Downs, Jos. Bates, John Ordronaux, John V. Holt, H. C. Gray, M. M. Wood, E. W. Bottum, John P. Gray, Austin White, Alex. Ayer, P. P. Staats, Wm. H. Bailey, E. M. Carmichael, M. L. Finch, —— Wolcott, Jas. H. Curry, John H. Reynolds, S. H. Freeman, M. H. Colby, J. K. Leaming, O. M. Allaban, P. McNaughton, Jas. Thorn, Avery Cook, Jas. M. Minor, J. N. Northrop, L. Barton, Wm. M Smith, E. W. Howard, J. V. Lansing, H. B. Salmon, Wm. Rockwell, Jacob Hant, E. S. F. Arnold, I. Botsford, S. R. Percy, J. G. Beckwith, M. C. Edmonds, John Pindar, H. F. Stevens, Oliver White, Jos. C. Hutchinson, I. E. Casey, T. B. Reynolds, J. G. Snell, Henry L. Sabine, C. R. Agnew, Peter Faling, J. H. Wheeler, M. F. Cogswell, Douglas Bly, Chas. A. Lee, J. V. P. Quackenbush, P. Van O'Linda, H. O. Carrington, Jas. McNaughton, Israel Parsons, Wm. F. Carter, Thos. Hun, S. W. Butler, L. A. Sayre, Wm. McCullum, L. G. Warren, J. R. Bontware, H. G. Bigelow, M. L. Meade, J. S. Mosher. J. E. Smith, J. M. Sturdevant, W. H. Craig, F. L. V. Chapin, Gurdon Buck, J. F. Flint, Jos. Lewis, Cyrus Ramsay, Staats Winne, W. C. Anderson, Chas. S. Tripler, Edward Duffy, Levi Moore, S. G. Delamater, Alex. Ennis, W. W. Greene, A. M. Smith, M. H. Burton, D. M. Devendorf, S. W. Greene, J. M. Delamater, Henry Marsh, W. H. Richardson. M. W. Burns, Ashael Perry, Archibald Gow, H. L. Saburn, J. R. Preston, H. S. Case, E. R. Seguin, Daniel Maybern, A. N. Gunn, Geo. W. Little, Thomas E. Burtsell, Chas F. Taylor, L. G. Warren, F. S. Lowe, Levy McLeane, B. S. Catlin, Asher Wright, H. B. Mayhen, Geo. W. Barr, W. G. Bigelow, A. Calkins. Making a representation of 166.

